# Stomatal development and epiphytic bacteria in sunflower hypocotyls[Author-notes fn0001]

**DOI:** 10.1080/15592324.2025.2548312

**Published:** 2025-08-23

**Authors:** Ulrich Kutschera, Rajnish Khanna

**Affiliations:** a I-Cultiver, Inc., Manteca, CA, USA; b Department of Plant Biology, Carnegie Institution for Science, Stanford, CA, USA

**Keywords:** Darwin, stomata, evolution, development, sunflower, cell elongation

## Abstract

Francis Darwin (1848–1925) was one of the first to study in detail the development and function of stomata. However, since the green leaves of flowering plants are the most conspicuous and suitable organs for the study of cell development and transpiration activity, most of this “Darwinian” research has focused on these sun-exposed organs. In this study, we analyzed the development of stomata along the hypocotyl of 5-day-old light-grown sunflower (*Helianthus annuus*) seedlings. We document a positive correlation between cell density and DNA content along the axial organ, and the step-by-step development of fully functional stomata in the meristematic region. However, along the stem, from tip to base, the size of the guard cells remained largely constant, whereas the epidermal cells in the neighborhood of the guard cells elongate by 100%, and those farther away by almost 300%. We suggest that symplastic isolation of dumbbell-shaped guard cells is responsible for this lack of growth, and that an inhibitory effect of the guard cells is involved. In addition, we discover and describe epiphytic bacteria on some of the epidermal cells investigated. The role of these microbes, as commensals or symbionts, is unknown.

## Introduction

In 1916, Francis Darwin (1848–1925) published a remarkable research article, wherein he described the relationship between stomatal movements and the transpiration activity of the leaves in a variety of plant species.^1^ As outlined in a recent biohistorical account,^2^ the son of Charles Darwin (1809–1882) was a Reader in Plant Physiology at Cambridge University and developed several novel methods for the study of the internal workings of the plant body. Among his inventions, Francis Darwin's mass flow porometer was the most spectacular, because, using green leaves, it became possible to quantify water loss via transpiration activity as a function of light.^3^

Since Darwin's time, the leaves became the model organism for the study of stomatal development, and *Arabidopsis thaliana* is one of the best-studied organisms.^4^ However, it has already been documented in detail by nineteenth-century botanists that not only the leaves but also the stems and other areal organs are characterized by stomata that permit the uptake of carbon dioxide and an accelerated release of molecular oxygen. In addition, transpiratory water loss may also be of importance in the stem and other plant organs.^3^

When Francis Darwin was a postdoc in the laboratory of the German botanist Julius Sachs (1832–1897), who is regarded as the founding father of plant physiology,^5^ sunflower seedlings were already well-established model organisms for the study of organ development,^6^ phototropic solar tracking, etc. This model system was used over the decades to elucidate the effects of light and plant hormones, such as auxins and gibberellins, on cell elongation. However, despite many insights, the morphology and development of stomata with respect to the standard epidermal cells have been largely ignored (reviewed in Kutschera and Niklas).^7^ Moreover, although epiphytic microbes, notably of the genus *Methylobacterium*, have been characterized on the cotyledons, leaves, and root system of sunflower seedlings and adults,^8^ no data are available as to a possible occurrence of bacteria on the hypocotyl of this economically important crop species.

The aim of this study was to analyze the cell-growth patterns along the stem, stomatal development, elongation of all types of peripheral cells, and the possible occurrence of epiphytic bacteria. We document that the guard cells maintain their size along the stem, whereas the “standard epidermal cells” that surround these specialized structures elongate.

## Materials and methods

Seeds (achenes) of sunflower (*Helianthus annuus* var. Giganteus) were planted in moist vermiculite and raised in a 12 h dark/12 h white light-regime, under a 150 µmol m^−2^ s^−1^ growing scheme as described.^7^ Five-day-old seedlings of average size, hypocotyl lengths 4–5 cm, were selected for all the experiments. For one set of studies, the cotyledons were excised and analyzed as described below. For all other experiments, the hypocotyl was cut below the onset of cotyledons and above the onset of the root system. Cell density and DNA content measurements of 5 mm-sections along the stem were carried out as described by Kutschera and Niklas.^7^ Stomatal development in the meristematic region was documented by using ca. 0.5 mm thin surface sections removed from the upper 5 mm region of the hypocotyl, employing bright-field microscopy. Immediately after cutting, the surface sections were fixed in 100% ethanol and stored before examination.

The dimensions of three types of epidermal cells were quantified using the “Cucumber-fixed-epidermis-technique” as described by Gibson et al.^9^ In our study, we distinguish between guard cells, epidermal cells 1 (surrounding guard cells), and epidermal cells 2 (at least three cells distant from guard cells and epidermal cells 1). Scanning electron micrographs (SEMs) of the epidermal surface in the meristematic region were prepared as described by Kutschera.^10^

## Results

### Growth pattern along the hypocotyl

**Figure 1.**
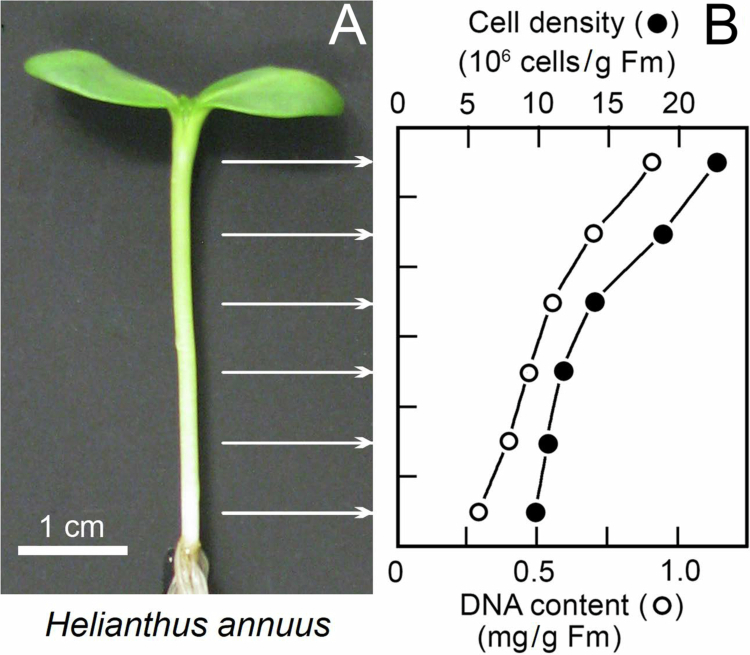
Photograph of a representative 5-day-old light-grown sunflower seedling. The six regions along the hypocotyl analyzed are indicated with white arrows (A). The cell density and DNA-content, determined in 5 mm-sections cut along the stem, both decreased from the top to the bottom of the axial organ (B). (the data represent the means of 12 measurements each; the S.E.M. values are not larger than the symbols).

In the first set of experiments, we analyzed the density of cells from the meristematic region (0–5 mm below the onset of the cotyledons) via sections to the base of the hypocotyl in 5-day-old de-etiolated (green) seedlings of *H. annuus* (Figure 1A). The hypocotyl was separated into six regions, 5 mm in length each. As Figure 1B shows, the cell density decreased from ca. 25 to about ten million cells/g fresh mass along the length of the hypocotyl, from top to bottom, with the steepest decline in the upper third of the organ. The DNA content decreased in parallel from ca. 0.9 to about 0.3 mg per g of fresh mass (the difference: ca. 2.5-fold decrease in cell density versus ca. 3.0-fold decrease in DNA-content per g fresh mass was not statistically significant). These data provide independent evidence for our previous finding that the region of cell division and elongation is largely restricted to the upper part of the stem.^7^ Since the meristematic region in 5-day-old etiolated sunflower seedlings is restricted to the upper 3 mm, we focused on this part of the stem in our attempt to document stomatal development using bright-field microscopy.

### Stomatal development in the meristematic region

**Figure 2.**
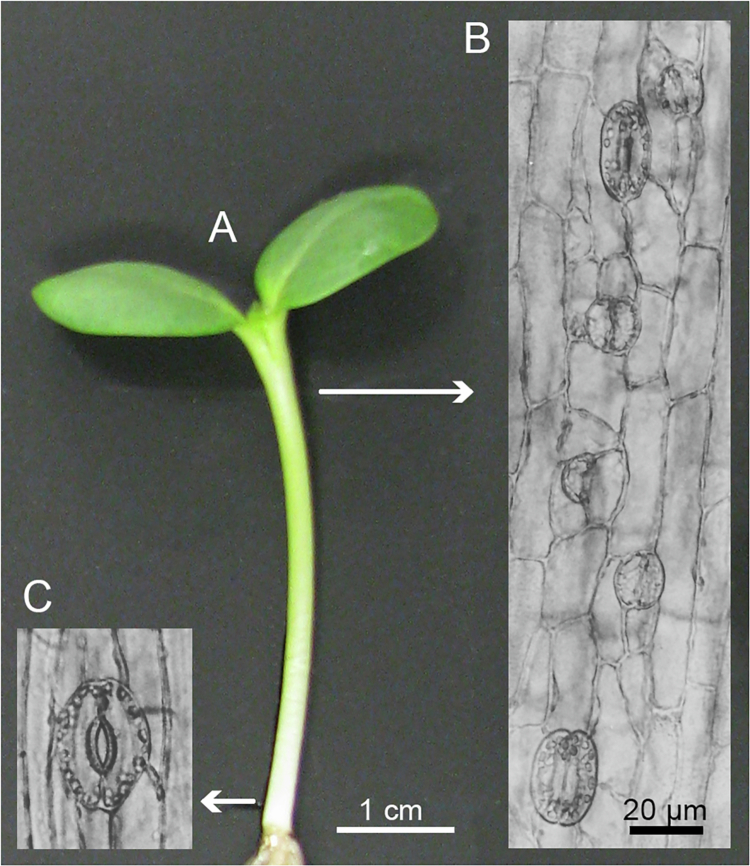
Stomatal development documented in a 5-day-old light-grown sunflower seedling (A). The light micrographs of fixed epidermal peels taken from apical vs. basal samples document three developmental stages: meristemoid mother cell (1), young (2) and mature stomata (3) (apical) (B); a representative fully developed, open stoma (basal) is shown in (C).

In these series of experiments, we focused on the apical (meristematic) and basal (mature) 5-mm-region of de-etiolated sunflower seedlings (Figure 2A). In the representative light micrographs depicted in Figure 2B and C, the development of functional stomata was observed. In the apical (meristematic) region, the development of juvenile (closed) stomata has been documented: meristemoid-mother cell, unequal cell division, and pairs of small guard cells are shown in Figure 2B. In the basal region, fully developed, open stomata, i.e., two guard cells with chloroplasts, are apparent (see Figures 2C and 4b). This indicates that in the mature region of the de-etiolated sunflower hypocotyl, the pores actively regulate gas and water exchange. Moreover, Figure 2B indicates that, in the epidermal layer, three types of cells can be distinguished: pairs of guard cells; short epidermal cells that surround the stomata; and much longer “typical” epidermal cells that represent the majority of cells of the outer tissue layer of the sunflower stem.

### Differential cell elongation along the stem

**Figure 3.**
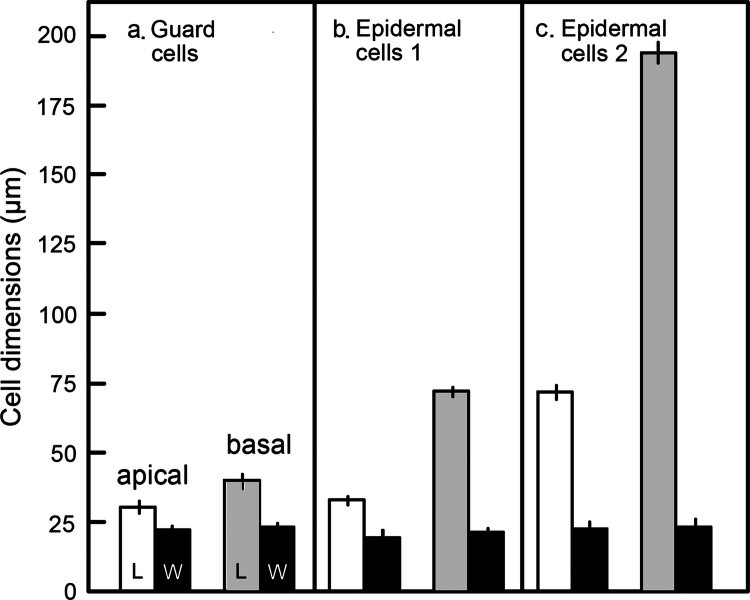
Cell dimensions in the apical and basal regions of 5-day-old light-grown sunflower hypocotyls (see Figure 2). Guard cells (stomata) (a), epidermal cells-1 in the vicinity of the stomata (b), and “typical” epidermal cells-2 in areas of the epidermal layer where stomata are absent (c). L = length, W = width of the cells (the data represent means ± S.E.M. of 100 measurements from five hypocotyls each).

**Figure 4.**
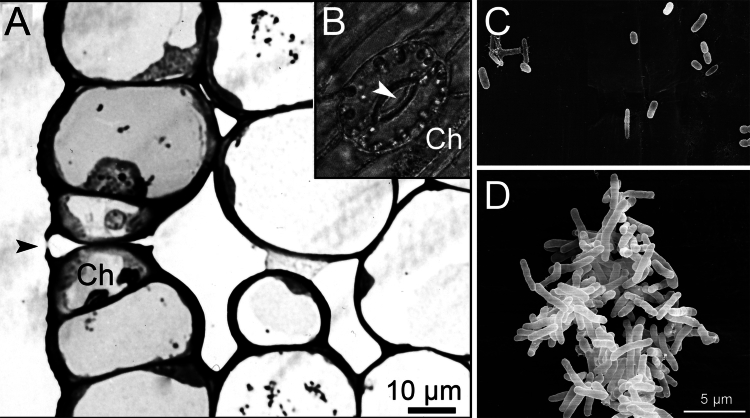
Light micrograph of the outer three cell layers (epidermis, subepidermis 1–2), taken from a longitudinal section through the middle region of a sunflower hypocotyl (A). The closed pore of a stoma is indicated by an arrowhead. A mature stoma with an open pore and chloroplasts (Ch) is shown in (B). On the epidermis of the apical part of the stem, epiphytic bacteria were observed and documented via representative REM images. Single bacteria (C) and clusters of microbes (D) were discovered in the meristematic region of the developing organ.

In the next set of experiments, we quantified the lengths and widths of these three types of cells in the apical and basal regions of the axial plant organ (Figure 3). In accordance with our light micrographs (Figure 2B,C), the guard cells in the basal region were only ca. 15% longer than those in the meristematic part of the organ. Hence, cell elongation along the growing stem was largely suppressed in these specialized pairs of peripheral cells (Figure 3). In contrast, the small epidermal cells elongated by ca. +100%. However, the largest growth along the stem was recorded in the “typical” epidermal cells (ca. +290%). This result indicates that the further away the cells are from the essentially nonelongating guard cells, the larger is their rate of elongation as the stem matures. Finally, Figure 3 shows the average widths of all three types of cells. Values of 20 to 25 micrometers were measured in all epidermal cells analyzed here. Hence, no significant increase in width took place along the growth gradient. In other words, cell expansion in the sunflower hypocotyl is anisotropic. Figure 4a shows the structure of a typical, mature stoma in the apical region of the sunflower hypocotyl. Chloroplasts are visible in the guard cells, organelles that are lacking in the surrounding epidermal cells of the mature zone of the stem (Figure 4b).

### Epiphytic bacteria in the apical region

In addition to the cell-dimension measurements reported above, we used scanning transmission microscopy (TEM) to corroborate our cell dimension-observations. In this context, we observed single microbes or clusters of epiphytic bacteria on the epidermal cells in the apical region of the stem (upper 5 mm). Figure 4c,d shows two representative images. We suggest that these bacteria are members of the extensively studied populations of methylobacteria observed on the cotyledons and primary leaves of sunflower seedlings and in the phyllosphere of bryophytes (such as the taxa *Methylobacterium marchantiae* and *M. funariae*; see ref. 18). These microbes consume methanol emitted by meristematic cells characterized by high metabolic activity. In the basal region of the hypocotyl, no epiphytic microbes were detected. This finding is in accordance with the fact that, after cessation of cell elongation, metabolic cell activity, and hence, methanol release is much lower than in the apical part of the developing organ.^7^^,^^8^

## Discussion

Francis Darwin was one of the pioneers with respect to the experimental analysis of development, structure and function of stomata in the “Kingdom Plantae.” As described in detail by Kutschera and Baluska,^2^ he invented novel methods and apparatuses to quantify the flow of water, from moist soil via stem and leaf tissue through open stomatal pores (transpiration stream). One of F. Darwin's basic insights was his 1916-hypothesis that, during daytime hours, water flow is regulated via opening and closing of the stomatal pores of the leaf. This concept, as well as other “Darwinian hypotheses” concerning stomatal function, later proved to be correct.^11^^,^^12^

In the most recent review on the topic of the present study entitled “Stomatal development in the changing climate,” Chua and Lan summarized our current knowledge as to the formation of stomata in the epidermal cell layer of plant organs. In accordance with the “evolutionary view” of Francis Darwin, Chua and Lan^13^^,^^14^ remark that the basic shape of stomata, as documented in ca. 400 million-year-old fossilized land plants, has hardly changed compared with their counterparts observed in extant embryophytes. Hence, the form and function of stomata may have been optimized soon after the origin of land plants, so that only stabilizing selection was active on these structures.^15^ In other words, stomata may be interpreted to consist of “living fossil cells,” but more work is required to corroborate this speculation.

In their *Review* on stomatal development, the authors refer to studies where *Arabidopsis thaliana* and several crop species were analyzed (cabbage, maize, rice, soybean, tobacco, tomato, sorghum, and wheat). Sunflower is not mentioned, despite the fact that this important crop species displays “phototropic solar tracking” in the upper part of the developing cormus, inclusive of the first leaves.^6^

Moreover, only studies on leaf tissue are mentioned, although it has long been known that the hypocotyl is likewise equipped with these dumbbell-shaped pairs of guard cells that surround a “stomatal pore.” To the best of our knowledge, this is the first study to focus on stomatal development in the developing sunflower stem. Our results show that cell density and DNA-content per g fresh mass are highest in the upper 5 mm-section of the stem, where the meristem is localized.^7^ In this region, we observed the development of stomata, which conforms with the pattern documented in *Arabidopsis* and many crop species.^13^^,^^16^ During organ growth, cell dimensions in the stomata-population of the epidermal cell layer remained largely constant, whereas cells close to the guard cells elongated by ca. **100%**. However, the *“***typical**” epidermal cells, which represent more than **95%** of the cell population in this outer tissue layer, are elongated by almost **300%**. We suggest that the lack of cell growth of the guard cells is caused by symplastic isolation, so that the hormonal signals that regulate elongation growth (auxin and other phytohormones) cannot reach these specialists for the regulation of water and gas exchange.

 The reason why the epidermal cells close to the stomata display a much smaller elongation response than the bulk of the “**typical**” epidermal cells is not known. We suggest that the guard cells emit an “inhibitory factor,” and that, to compensate the reduced elongation response, these cells also divide during organ growth. However, direct evidence to support this hypothesis is lacking. At any rate, the discovery of the fact that we have to distinguish between two types of epidermal cells that elongate at much different rates documents that organ growth is more complex than we have previously thought.

Finally, our study shows that, in the meristematic region of the *Helianthus*-stem, epiphytic microbes are present. These bacteria, which exist as single cells or clusters (Figure 4c,d), were not observed in the basal region of the organ. We suggest that most of these microbes represent phytohormone-secreting methylotrophic bacteria that consume methanol emitted by meristematic cells (methanol is produced via the action of pectin methyl esterases during cell wall metabolism, see refs).^17-19^ Since the meristem has the largest metabolic activity (see Figure 1), it is likely that the putative methylobacteria feed on the released metabolites of the epidermal cells. These side products of wall metabolism are largely lacking in the fully elongated cells at the base of the stem.

In summary, our detailed study of the cell populations in the epidermal layer of the sunflower stem revealed that the classical distinction between “epidermal cells” and “stomata,” the former elongating, the latter remaining in approximately the same size, is too simplistic. We found three classes of cells in the epidermis that display different cell elongation patterns as the stem matures. More work is required to determine whether this novel finding is restricted to sunflower, using the hypocotyls of other crop plants as experimental material. In addition, it is obvious that the hormonal regulation of stem elongation is more complex than we have previously assumed.
